# 

**Published:** 1891-08

**Authors:** 


### Communications.

### NATIONAL ASSOCIATION OF DENTAL FACULTIES.

[From proof sheets kindly furnished by *Dental Cosmos.*]

The eighth annual meeting of the National Association of Dental Faculties was held at Saratoga Springs, commencing Saturday, August 1, 1891; the president Dr. L. D. Carpenter, Atlanta, in the chair.

The following colleges were represented at the meeting:

*Baltimore College of Dental Surgery.*—R. B. Winder.

*Boston Dental College.*—John A. Follett.

*Chicago College of Dental Surgery.*—Truman W. Brophy.

*Harvard University, Dental Department*—Thomas Fillebrown.

*Kansas City Dental College.*—J. D. Patterson.

*Missouri Dental College.*—Wm. H. Eames.

*New York College of Dentistry.*Frank Abbott.

*Ohio College of Dental Surgery.*—H. A Smith.

*Pennsylvania College of Dental Surgery.*—C. N. Peirce.

*Philadelphia Dental College.*—Henry I. Dorr.

*State University of Iowa, Dental Department.*—A. O. Hunt.

*University of Michigan, Dental Department.*—J. Taft.

*University of Pennsylvania, Dental Department.*—James Truman.

*Vanderbilt University, Dental Department.*—W. H. Morgan.

*Louisville College of Dentistry.*—James Lewis Howe.

*Indiana Dental College.*—Junius E. Cravens.

*University Dental College.*—George H. Cushing.

*Dental Department of National University.*—James B. Hodgkin.

*Dental Department of Southern Medical College.*—L. D. Carpenter.

*School of Dentistry of Meharry Medical Department of Central Tennessee College.*—G. W. Hubbard.

*University of Maryland, Dental Department.*—Isaac H. Davis.

*Royal College of Dental Surgeons of Ontario.*—J. B. Willmott.

*American College of Dental Surgery.*—T. Clendenen.

Dr. H. B. Noble, of Columbian University Dental Department, was also present, but not as a delegate.

The University of Denver, Dental Department, was reported upon favorably by the Executive Committee, and was elected a member of the association, and its representative, W. F. McDowell, took part in the meeting.

The application of Western Dental College, Kansas City, and Dental Department Medical College of Tennessee, Knoxville, were reported favorably, and under the rules laid over for one year.

On motion, it was directed that the National Association of Dental Examiners be requested to appoint a committee of five to confer with a similar committee from the National Association of Dental Faculties in regard to matters of mutual interest between the two associations, their conclusions to be reported back to this meeting. The president appointed as the committee on behalf of the Faculties Association, Drs. Peirce, Winder, Eames, Truman and Morgan.

The committee subsequently reported the following resolutions as having been agreed to by the conference committees, and recommended that they be confirmed, which was accordingly done:

*Resolved*, That it is recommended to the State boards that when a graduate after examination has been refused a license and his college requests information as to the causes of his failure to pass the examination, the boards shall furnish the faculty with a detailed statement of the subjects and questions on which the applicant has failed.

*Resolved*, That we discountenance the publication by the State boards of the names of colleges whose graduates have failed to pass.

The committee also reported favorably a communication from the National Association of Dental Examiners, which have been referred to them, as follows:

"As the next session of the colleges marks the commencement of the new plan of three years' college instruction, we recommend that this association request the National Association of Dental Faculties to require each school to issue each year an announcement containing a list of the students classified in the three grades of seniors, juniors, and freshmen; that this list also in each instance designate the absentees, and that each school be required in the same announcement to publish a list of the graduates of the preceding sessions."

On motion of Dr. Cushing, the term absentee in the foregoing was construed to mean one who for any reason has not attended a full course.

The president being asked to rule upon the resolution adopted last year requiring dissections, decided that it was mandatory.

The following, offered by Dr. Abbott, was adopted by a unanimous vote:

*Resolved*, That any college whose regularly appointed representative fails to sign the constitution within one year from the time of its election to membership shall be dropped from the roll of membership of this association.

Dr. Patterson, from the committee on codification of the rules, presented a report, which was amended and adopted, as follows:

ENTRANCE.

*Resolved*, That a preliminary examination be required for entrance to our dental colleges. Such requirements shall include a good English education. In case of any applicant failing to pass a satisfactory preliminary examination, the other colleges of this association may be informed of the fact. (Saratoga, 1884; Chicago, 1885.)

*Resolved*, That a candidate for matriculation who presents a diploma from a reputable literary institution, or other satisfactory evidence of literary qualifications, shall be admitted without further examination. Saratoga, (1884; Chicago, 1885.)

*Resolved*, That after the session of 1890-91 a diploma from a reputable medical college shall entitle its holder to enter the second course in dental colleges of this association, but he may be excused from attendance upon lectures and examinations upon the following subjects: General anatomy, chemistry, physiology, materia medica and therapeutics. (Excelsior, 1890.)

### ADMISSION TO SENIOR CLASS.

*Resolved*, That the colleges of this association may receive into the junior or senior classes only such students as hold certificates of having passed a satisfactory examination in the studies of the freshman or junior years respectively; this certificate to be a pledge to any college to which they may apply that a previous year has been properly spent in the institution. (Chicago, 1883; changed, Saratoga, 1891.)

*Resolved*, That applicants for admission to advanced standing from foreign countries shall be required to furnish properly attested evidence of study, attendance upon lectures, etc., the same as required of applicants here; and they shall pass the intermediate examinations. (Chicago, 1885; changed, Saratoga, 1891.)

### FORM OF INTERMEDIATE CERTIFICATE.

Place——————Date——————

This certificate that——————has been a member of the ——————class in the——————————college during the session of————.

He was examined at the close of the session in the studies required (as follows), and is entitled to enter the ——————class.

Attendance——————

| *Freshman Year.* | *Junior Year.* |
| --- | --- |
| *a*—————— | *a*—————— |
| *b*—————— | *b*—————— |
| *c*—————— | *c*—————— |
| *d*—————— | *d*—————— |
| *e*—————— | *e*—————— |

Each student completing a regular course in any college of this association must be furnished with the above certificate, without which he shall not be accepted by any college for admission to the advanced class, except by conference with and consent of the school from whence he came. (Chicago, 1885; changed, Saratoga, 1891.)

*Resolved*, That the dean of each school shall upon request furnish the executive committee with the exact character of the intermediate examination held in his school, and whether or not the examination is final. (Niagara, 1886; changed, Saratoga, 1891.)

ATTENDANCE.

*Resolved*, That candidates for admission to the colleges of this association, who are undergraduates in a reputable medical college, may be admitted to the junior class subject to the rules of examination governing admission to that class.

*Resolved*, That no college shall give credit for a full term to any student who has entered later than twenty days after the beginning of regular lectures. (Chicago, 1885.)

*Resolved*, That no college of this association shall admit a student after twenty days from beginning of regular term, except those colleges having a term of more than five months, and that they shall have an extension of time in proportion to the length of tarm. (Niagara, 1886.)

*Resolved*, That attendance upon three full regular courses of not less than five months each in separate years shall be required before examination for graduation. (Saratoga, 1889.)

GENERAL.

*Resolved*, That the fees of all dental colleges, as far as possible, be uniform. (Chicago, 1885.)

*Resolved*, That the members of any faculty belonging to this association may take part in its discussions, but only the delegated representatives shall vote upon a question before the association. (Niagara, 1886.)

*Resolved*, That dental schools which do not conform to the regulations of this association shall not be recognized by the association. (Niagara, 188 .)

*Resolved*, That a standing committee on schools be elected, whose duty it shall be to ascertain as far as practicable the workings of all dental schools in this country and

Europe, and be required to furnish information to the dean or secretary of any college when desired, and to report in writing at each meeting of this association. (Niagara, 1886.)

*Resolved*, That we agree to adopt a graded course of instruction and an intermediate examination between each course, which course of instruction and examination shall be conducted as the faculties of the different colleges represented in this association may deem proper. (Saratoga, 1884; changed, Saratoga, 1891.)

*Resolved*, That no charges against any faculty shall be reported to the association by any committee before both parties interested have been notified, and an opportunity given for a hearing before said committee. (Washington, 1887.)

*Resolved*, That as a matter of courtesy, when a student leaves one school to go to another, the dean of the second college shall write to the dean of the first college inquiring whether there may be any objections to the transfer; this to be done whether the student presents a certificate or not. (Louisville, 1888; changed, Saratoga, 1891.)

*Resolved*, That hereafter a delegate representing a college in this association shall be a member of a teaching faculty, and shall present credentials from the college to which he belongs, legally authorizing him to represent his college before he shall be entitled to vote. (Louisville, 1888.)

*Resolved*, That it shall be obligatory upon the dental schools belonging to this association to publish the names of all their matriculates and graduates of the preceding session, with the states and countries from which they come, in their regular annual announcement, and that an asterisk (*) accompany the name of each person not in attendance, and the words "not in attendance" be placed as an explanatory foot-note at the bottom of the page.

*Resolved*, That the degree of Doctor of Dental Surgery shall not be conferred by any college belonging to this association honorarily, except by the consent of this association. (Saratoga, 1889.)

*Resolved*, That the term anatomy shall be interpreted to include didactic and practical anatomy, and that in the latter at least two parts of the cadaver shall be dissected in some regularly appointed anatomical department. (Excelsior, 1890.)

MEMBERSHIP.

*Resolved*, That all applications for membership in this association shall be made in writing, and referred to the executive committee. (Niagara, 1886.)

*Resolved*, That applicants for membership in this association shall be regularly incorporated dental colleges or departments of medical colleges or universities, wherein at least one full course of lectures has been delivered, and that such dental colleges or departments shall have been in existence at least one scholastic year. (Washington, 1887.)

*Resolved*, That no application for membership in this association be reported to the executive committee, unless received at least sixty days before the regular meeting. Washington, 1887.

*Resolved*, That all applications for membership reported upon favorably by the executive committee shall lie over one year before final action may be taken thereon. (Saratoga, 1889.)

*Resolved*, That we recommend that students take two full courses in studies of a general character, such as anatomy, physiology, chemistry, general principles of surgery, materia medica, and therapeutics, and three courses in those of a special dental character. (Excelsior, 1890.)

*Recommended*, That for a full course of lectures the minimum sum of college fees be $100.00. That diploma fees may be omitted, and an examination fee of not less than $25.00 be substituted therefor and made non-returnable; that a matriculation fee of $5.00 be charged annually. Special-course fees to be $10.00 for each branch taken, and $5.00 matriculation fee.

The American College of Dental Surgery, Chicago, was suspended from membership for two years for violation of the rules of the association in accepting students after the prescribed time and giving them credit for a full term.

A resolution dismissing charges against the Philadelphia Dental College was adopted.

The following were offered and laid over for one year under the rules.

By Dr. Hunt:

*Resolved*, That in case of charges against any college no final action shall be taken until all parties concerned shall have had at least thirty days' notice.

By Dr. Truman:

*Resolved*, That at all future meetings of the National Association of Dental Faculties the delegates shall consist of members of faculties, and demonstrators will not be received.

By Dr. Fillebrown:

*Voted*, That after June, 1893, the yearly course of study shall be not less than seven months, two months of which may be attendance upon clinical instruction in the infirmary of the school, now known as intermediate or infirmary courses.

By Dr. Hunt:

*Resolved*, That after the session of 1892-93, four years in the study of dentistry be required before graduation.

Dr. Dorr offered a resolution that students who have successfully passed their examinations for advanced standing shall have their certificates given or mailed to them within thirty days after such examinations shall have been completed. Adopted.

The following officers were elected for the ensuing year: W. H. Eames, St. Louis, president; J. D. Patterson, Kansas City, vice-president; J. D. Patterson, Kansas City, Secretary; H. A. Smith, Cincinnati, treasurer; J. Taft, A. O. Hunt, and Frank Abbott, executive committee.

The president appointed the following standing committees: Ad Interim Committee, James Truman, Frank Abbott and Thomas Fillebrown; Committee on Schools, D. R. Stubbefield, J. A. Follett, J. Lewis Howe, J. E. Cravens, S. H. Guilford.

Adjourned to meet at the place of the next meeting of the American Dental Association, on the Monday previous, at 10 o'clock.

---

### NATIONAL ASSOCIATION OF DENTAL EXAMINERS.

[From proof sheets kindly furnished by *Dental Cosmos.*]

The National Association of Dental Examiners held its tenth annual session at Saratoga Springs, commencing Monday August 3, 1891.

In the absence of the regularly elected officers, Dr. L. D. Shepard was elected president, and Dr. Levy secretary-pro tem.

The following State boards of dental examiners were represented:

*Vermont.*—James Lewis.

*New Jersey.*—Fred. C. Barlow, Fred. A. Levy.

*Ohio.*—J. Taft, H. A. Smith, L. E. Custer, C. R. Butler.

*Pennsylvania.*—Louis Jack, W. E. Magill.

*Georgia.*—George W. McElhaney.

*Maryland.*—A. J. Volck.

*Massachusetts.*—L. D. Shepard, J. S Hurlbut.

*Mississippi.*—W. H. Marshall.

*Iowa.*—J. T. Abbott.

*Louisiana.*—Geo. J. Friedrichs.

The following State boards were admitted to membership:

*Tennessee.*—J. Y. Crawford.

*New Hampshire.*—E. B. Davis.

*Maine.*—E. J. Roberts, D. W. Fellows.

A committee consisting of Drs. Shepard, Fellows, Crawford, McElhaney and Magill was appointed to confer with a similar committee from the National Association of Dental Faculties, for the better understanding of questions involving educational interests.

The committee subsequently reported that the conference committees had agreed upon the following resolutions, which were, on motion, confirmed:

*Whereas*, There can be no question that the main object in view of both the National Association of Dental Faculties and the National Association of Dental Examiners is the better preparation of the young dentists for usefulness in the community, and that to secure this end it is desirable that the State boards of dental examiners and the colleges should work in harmony; therefore

*Resolved*, That it is recommended to the State boards that when a graduate after examination has been refused a license and his college requests information as to the causes of his failure to pass the examination, the boards shall furnish the faculty with a detailed statement of the subjects and questions on which the applicant has failed.

*Resolved*, That we discountenance the publication by the State boards of the names of colleges whose graduates have failed to pass.

The committee recommended that "as the next session of the colleges marks the commencement of the new plan of three year's college instruction, that this association request the National Association of Dental Faculties to require each school to issue each year an announcement containing a list of students, classified in the three grades of seniors, juniors and freshmen; that this list also in each instance designate the absentees, and that each school be required in the same announcement to publish a list of the graduates of the preceding year.

The committee on colleges reported that they had received reports of the number of matriculates and graduates of twenty-eight colleges, as shown below:

| NUMBER OF MATRICULATES AND GRADUATES OF THE DENTAL COLLEGES. | Matriculates. | Absentees. | Graduates. | Ratio. |
|---|---|---|---|---|
| Baltimore College of Dental Surgery, Baltimore, Md. | 224 | 3 | 76 | 34.3 |
| Boston Dental College, Boston, Mass. | 96 | | 31 | 32.2 |
| Chicago College of Dental Surgery. Chicago, Ill. | 323 | | 94 | 29. |
| Harvard University, Dental Department, Boston, Mass. | 44 | | 15 | 34. |
| Kansas City Dental College, Kansas City, Mo. | 110 | 5 | 43 | 40. |
| Missouri Dental College, St. Louis, Mo. | 90 | | 26 | 28.8 |
| New York College of Dentistry, New York, N. Y. | 283 | 8 | 85 | 30.9 |
| Ohio College of Dental Surgery, Cincinnati, Ohio. | 208 | | 75 | 36. |
| *Pennsylvania College of Dental Surgery, Philadelphia, Pa. | 252 | 17 | 94 | 40. |
| †Philadelphia Dental College, Philadelphia, Pa. | 315 | | 146 | 46.3 |
| University of California, Dental Dept., San Francisco, Cal. | 63 | | 16 | 25.4 |
| University of Iowa, Dental Department, Iowa City, Ia. | 161 | | 58 | 36. |
| University of Michigan, Dental Dept., Ann Arbor, Mich. | 132 | 1 | 29 | 22.1 |
| University of Pennsylvania, Dental Dept., Philadelphia, Pa. | 206 | 3 | 83 | 40.8 |
| Vanderbilt University, Dental Department, Nashville, Tenn. | 135 | | 43 | 31.8 |
| Northwestern College of Dental Surgery, Chicago, Ill. | 14 | | 3 | 21.4 |
| Indiana Dental College, Indianapolis, Ind. | 96 | | 40 | 41.6 |
| Dental Dept. of Southern Medical College, Atlanta, Ga. | 103 | | 38 | 36.8 |
| School of Dentistry of Meharry Medical College, Department of Central Tennessee College, Nashville, Tenn. | 5 | | 1 | 20. |
| University of Maryland, Dental Dept., Baltimore, Md. | 163 | 4 | 64 | 39.7 |
| Columbian University, Dental Dept., Washington, D. C. | 19 | | 2 | 10.5 |
| Royal College of Dental Surgeons of Ontario, Canada. | 67 | | 27 | 40. |
| College of Dentistry, Department of Medicine, University of Minnesota, Minneapolis, Minn. | 36 | | 7 | 19.4 |
| American College of Dental Surgery, Chicago, Ill. | 167 | | 49 | 29. |
| Aggregate | 3312 | | 1144 | 34.5 |

* 15 female matriculates. † 9 female matriculates.

| DENTAL COLLEGES NOT CONNECTED WITH THE NATIONAL ASSOCIATION. | Matriculates. | Absentees. | Graduates. | Ratio. |
|---|---|---|---|---|
| German-American, Chicago, Ill. | 22 | | 11 | 50. |
| Western Dental College, Kansas City, Mo. | 61 | 1 | 9 | 15. |
| United States Dental College, Chicago, Ill. | 43 | | 11 | 25.5 |
| College of Dentistry, University of Denver, Denver, Col. | 12 | | 5 | 41.6 |
| Aggregate | 139 | | 36 | |
| Whole aggregate | 3451 | | 1180 | |

Four colleges, members of the National Association of Dental Faculties, had failed to report, namely: University Dental College, Chicago; Dental Department, University of Tennessee; Louisville College of Dentistry; and Dental Department of National University, Washington, D. C.

The committee also reported the following list of colleges which they recommend as reputable:

Baltimore College of Dental Surgery, Baltimore, Md.

Boston Dental College, Boston, Mass.

Chicago College of Dental Surgery, Chicago, Ill.

College of Dentistry, Department of Medicine, University of Minnesota, Minneapolis, Minn.

Dental Department, Columbian University, Washington, D. C.

Dental Department of Northwestern University (now the University Dental College), Chicago, Ill.

Dental Department of Southern Medical College, Atlanta, Ga.

Dental Department of University of Tennessee, Nashville, Tenn.

Harvard University, Dental Department, Cambridge, Mass.

Indiana Dental College, Indianapolis, Ind.

Kansas City Dental College, Kansas City, Mo.

Louisville College of Dentistry, Louisville, Ky.

Missouri Dental College, St. Louis, Mo.

New York College of Dentistry, New York City.

Ohio College of Dental Surgery, Cincinnati, Ohio.

Pennsylvania College of Dental Surgery, Philadelphia, Pa.

Philadelphia Dental College, Philadelphia, Pa.

School of Dentistry of Meharry Medical Department of Central Tennessee College, Nashville, Tenn.

University of California, Dental Department, San Francisco, Cal.

* Northwestern College of Dental Surgery, Chicago (defunct).

University of Iowa, Dental Department, Iowa City, Ia.

University of Maryland, Dental Department, Baltimore, Md.

---

* The diplomas of this college are discredited after 1889.

University of Michigan, Dental Department, Ann Arbor, Mich.

University of Pennsylvania, Dental Department, Philadelphia, Pa.

Vanderbilt University, Dental Department, Nashville, Tenn.

Western Dental College, Kansas City, Mo.

Minnesota Hospital College, Dental Department, Minneapolis, Minn. (defunct).

St. Paul Medical College, Dental Department, St. Paul, Minn. (defunct).

The report was adopted, and the committee on schools was enlarged to consist of one member from each State board. Dr. Louis Jack was appointed chairman of the committee, and the president, secretary, and chairman were authorized to complete its membership and to fill vacancies.

The following was adopted:

*Resolved*, That this association requests the several boards represented in this body not to indorse beneficiary students.

The following officers were elected for the ensuing year: L. D. Shepard, president; W. E. Magill, vice-president; Fred. A. Levy, secretary-treasurer.

The officers were empowered to select the time and place of the next meeting.

Adjourned.

---

### MISSOURI STATE DENTAL ASSOCIATION.

The twenty-seventh annual meeting of the Missouri State Dental Association was held at Louisiana, Mo., July 7th to 10th inclusive. The following officers were elected for 1892: President, Dr. Geo. L. Shepard, Sedalia, Mo.; Vice-President, Dr. E. E. Shattuck, Kansas, City, Mo.; 2d Vice-President, Dr. J. T. Fry, Moberly, Mo; Cor. Secretary, Dr. Wm. Conrad, St. Louis, Mo.; Rec. Secretary, Dr. W. M. Carter, Sedalia, Mo.;

Treasurer, Dr. J. A. Price, Weston, Mo.; Board of Censors, Drs. C. J. McBride, Perryville; W. H. Buckley, Liberty; DeC. Lindsley, St. Louis, Mo.; Committee on Ethics, Drs. F. Slater, Rich Hill; E. B. Crane, California; E. W. Bear, Sedalia, Mo., Publication Committee, Drs. E. E. Shattuck, Kansas City; W. S. Lowry, Kansas City; W. E. Tucker, Springfield, Mo.; Committee on Law, Dr. J. A. Price, Weston, Mo; Committee on New Appliances, Dr. J. B. Vernon, St. Louis. The next meeting will be held at Clinton, Mo., the first Tuesday after July 4th, 1892.

WM. CONRAD,
Cor. Secretary.

---

### AMERICAN PUBLIC HEALTH ASSOCIATION.

The nineteenth annual meeting will be held at Kansas, City, Oct. 20th to the 24th, 1891. The Local Committee of Arrangements announces that all the Railway Passenger Associations of the county have granted a one and one-third fare rate for the round trip on the usual certificate plan, that is:

1. Procure a certificate of attendance from the agent at the starting point by paying full fare to Kansas City.

2. Have the certificate of attendance signed by the proper officer of the Association at Kansas City. This certiwill then procure return ticket for one third fare. All the leading hotels of Kansas City will give special rates to delegates. Arrangements are being perfected for an excursion into Kansas, as one of the features of the entertainment of the Association. For any information as to the meeting, address

Dr. E. R. LEWIS, Chairman,
or Dr. JOSEPH SHARP, Secretary, Local Com. of Arrangements, Kansas City, Mo.

---

THE TWENTY-FOURTH ANNUAL MEETING of the American Academy of Dental Science will be held in Boston on Wednesday, November 11, 1891, at 3 p. m. The annual address will be delivered by George S. Allan, D. D. S., of New York.

E. N. HARRIS,
*Cor. Secretary.*

248 Boylston St., Boston, Mass.

### MARYLAND STATE DENTAL ASSOCIATION.

The ninth annual meeting of the Maryland State Dental Association will be held in Baltimore at the St James Hotel, commencing Monday, December 14th at 8 p. m., and continuing through the 15th and 16th. Valuable papers will be read and exhibits made.

A cordial invitation is extended to members of the profession, and especially to those residing in the State of Maryland, to be present.

F. F. DREW, *Cor. Sec'y*,
701 N. Howard St.

### To Readers and Correspondents.

Communications intended for publication, and exchange journals and papers, should be addressed to the Editor of The American Journal of Dental Science, No. 845 N. Eutaw St. Baltimore, Md. All letters relating to the business of the Journal, or containing cash remittances, to be directed to Messrs. Snowden & Cowman, No. 9 W. Fayette Street, Baltimore, Md. Articles for publication should be received by the Editor at least two weeks previous to the first of the month on which the number in which it is designed for them to appear, is issued.

The Twenty-fourth Annual Meeting of the American Academy of Dental Science. - - - - - - - - 189

Maryland State Dental Association. - - - - - 190

### EDITORIAL.

Bones Ejected from Her Body. - - - - - - - 190

### MONTHLY SUMMARY.

How to Get a Tin Cast. - - - - - - - - 191

Aristol. - - - - - - - - - 192

☞ The American Journal of Dental Science will contain fifty pages of reading matter in each number, making a volume of about Six Hundred pages,

EXCLUSIVE OF ADVERTISEMENTS

